# Combined Letrozole and Growth Hormone Therapy in Late-Pubertal Males with Advanced Bone Age and Open Knee Physes

**DOI:** 10.3390/children13081077

**Published:** 2026-08-14

**Authors:** Hui Zhang, Pengxiang Zhou, Yunpu Cui, Xuemei Wang, Huiqiang Liu, Jinfang Yuan, Hua Zhang, Rongsheng Zhao, Tongyan Han, Xinli Wang

**Affiliations:** 1 Department of Pediatrics, Peking University Third Hospital, Beijing 100191, China; huizhang@bjmu.edu.cn (H.Z.);; 2 Department of Pharmacy, Peking University Third Hospital, Beijing 100191, China; pxzhou0427@bjmu.edu.cn (P.Z.);; 3 Institute for Drug Evaluation, Peking University Health Science Center, Beijing 100191, China; 4 Clinical Epidemiology Research Center, Peking University Third Hospital, Beijing 100191, China

**Keywords:** letrozole, growth hormone, bone age, final adult height, real-world study

## Abstract

**Highlights:**

**What are the main findings?**

- In this single-arm retrospective study, combined letrozole and growth hormone therapy was associated with a mean gain of 6.58 ± 3.46 cm in final adult height relative to baseline predicted adult height in late-pubertal males with BA 15 –< 18 years and open knee physes (*p* < 0.001).
- The observed height gain diminished with advancing baseline bone age; the largest gain (10.04 ± 4.66 cm) was seen in the BA 15 –< 16 subgroup, where final height exceeded mid-parental height.

**What are the implications of the main findings?**

- Knee epiphyseal patency may serve as an indicator of residual growth potential in adolescents with advanced wrist bone age, potentially broadening the therapeutic window for aromatase inhibitor plus GH therapy beyond conventional age thresholds.
- These preliminary findings suggest that baseline skeletal maturity, particularly knee physeal status, should be considered when individualizing treatment decisions; however, prospective controlled studies are needed to confirm these observations.

**Abstract:**

**Background/Objectives:** The therapeutic window for growth hormone (GH) therapy in late-pubertal males with advanced bone age (BA ≥ 15 years) is generally considered closed. Yet, open knee physes may indicate residual growth potential. This single-arm retrospective study evaluated the efficacy and safety of letrozole combined with GH in Chinese males with short stature, advanced BA (15 –< 18 years), and open knee physes. **Methods:** We included 139 male adolescents stratified by baseline BA: 15 ≤ BA < 16 (*n* = 24), 16 ≤ BA < 17 (*n* = 31), and 17 ≤ BA < 18 (*n* = 84). All received subcutaneous GH (0.05–0.07 mg/kg/day) and oral letrozole (2.5 mg/day). The primary outcome was the change in final adult height (FAH) from baseline predicted adult height (PAH). Longitudinal changes in height velocity and height SDS-BA were also assessed. **Results:** Among 75 patients who reached FAH, combination therapy was associated with a mean gain of 6.58 ± 3.46 cm over baseline PAH (*p* < 0.001). The gain was inversely correlated with baseline BA; the largest gain occurred in the 15 ≤ BA < 16 subgroup (10.04 ± 4.66 cm), significantly exceeding gains in the 16 ≤ BA < 17 (6.30 ± 3.01 cm) and 17 ≤ BA < 18 (5.70 ± 2.52 cm) subgroups (*p* < 0.001). In the youngest subgroup, FAH exceeded mid-parental height. The safety profile was acceptable; acne was the most common adverse event (46.76%), and no severe metabolic or endocrine disorders were observed. **Conclusions:** Letrozole plus GH therapy may be associated with improved FAH in late-pubertal males with advanced BA and open knee physes, with gains varying by baseline skeletal maturity and being numerically greatest in those with BA 15 –< 16 years. Knee epiphyseal status may be a useful consideration when individualizing therapy in this population.

## 1. Introduction

The management of short stature in late-pubertal males with advanced bone age (BA 15 –< 18 years) and open knee physes remains a clinical challenge in pediatric endocrinology. While final adult height (FAH) gain remains the primary goal, therapeutic interventions confront three biological barriers: rapid growth plate fusion, shortened linear growth windows, and variable responses to growth hormone (GH) therapy [1,2]. In Chinese populations, the median height-for-age plateaus at approximately 15 years, with residual growth potential diminishing to 2–4 cm before complete cessation by 18 years—a trajectory that appears earlier than in some Western populations, where growth may persist until later adolescence [3,4]. This compressed growth phase narrows the therapeutic window for GH in BA ≥ 15 males [5].

Effective therapy requires dual modulation of estrogen-mediated epiphyseal closure (via aromatase inhibition) and GH/insulin-like growth factor-1 (IGF-1) axis activation. While gonadotropin-releasing hormone analogs (GnRHa) combined with GH can prolong growth periods, their pubertal suppression, metabolic risks, and cost limit utility in late puberty [2,6]. Aromatase inhibitors (AIs) offer a targeted alternative by suppressing estrogen synthesis, elevating testosterone, and delaying growth plate fusion [7]. Both anastrozole (1 mg) and letrozole (2.5 mg) achieve near-complete aromatase inhibition (96.7% and >99.1%, respectively) [8]. Clinical evidence spanning two decades suggests that AI monotherapy increases FAH by 4–5 cm, while combined AI-GH therapy can increase gains to ~9 cm over ≥2 years [2]. Safety profiles from available studies appear generally favorable, with no consistent adverse impacts reported on insulin sensitivity, lipids, or sperm parameters [9,10,11,12].

Current clinical guidance has largely focused on AI-GH therapy in patients with BA < 14.5 years, with limited data available for those with BA ≥ 15 years due to concerns about exhausted growth potential [2,13]. However, this threshold may not fully account for regional variability in growth plate maturation: while wrist physeal closure typically concludes by BA 18 years, knee epiphyseal fusion can persist until 15.9–21.7 years—a potentially stronger predictor of residual growth [14]. Given that lower limbs contribute more than 50% of pubertal height gain, preserved knee physeal patency in late-pubertal males may indicate untapped growth potential despite advanced wrist maturation [15,16]. Thus, AI-GH therapy may offer a synergistic approach to delay epiphyseal closure and support linear growth, even in patients with BA 15 –< 18 years.

This study leverages real-world data from Chinese adolescents to address two questions: (1) In males with BA 15 –< 18 years and open knee physes, is letrozole-GH combination therapy associated with FAH gains? (2) What is the safety profile of this regimen in this population? Treatment decisions were made in routine clinical practice and were not randomized. By analyzing decadal clinical outcomes, we aim to provide real-world evidence to inform individualized treatment considerations in this understudied population.

## 2. Materials and Methods

### 2.1. The Materials Study Design and Participants

This single-arm, retrospective study included male adolescents (chronological age 12–18 years) with short stature, advanced bone age (BA 15 –< 18 years), and open knee epiphyses who were treated at Peking University Third Hospital between 2013 and 2023. Treatment was not randomized; all therapeutic decisions were made by treating physicians in routine clinical practice. Participants were identified through systematic review of growth charts and medical records.

Inclusion criteria were: (1) height SDS ≤ −2 or predicted adult height (PAH) SDS ≤ −2, or PAH below mid-parental height (MPH); and (2) ≥3 months of combined letrozole and GH therapy. Exclusion criteria included chromosomal or chronic systemic diseases, prior exposure to GnRHa, AIs, or GH, and confirmed GH deficiency (ruled out by GH stimulation testing when suspected based on slow growth velocity or subnormal serum IGF-1).

A total of 139 males were included in the baseline analysis. Among patients with a clear short-stature diagnosis, the distribution was: idiopathic short stature (*n* = 48, 34.5%), familial short stature (*n* = 18, 12.9%), constitutional delay of growth and puberty (*n* = 10, 7.2%), and small for gestational age (*n* = 9, 6.5%). The remaining 54 patients (38.8%) were enrolled primarily because their predicted adult height fell below mid-parental height and were not assigned a specific diagnosis. Of these, 75 had follow-up data available for FAH assessment. Participants were stratified into three subgroups based on baseline BA: 15 ≤ BA < 16 years (*n* = 24), 16 ≤ BA < 17 years (*n* = 31), and 17 ≤ BA < 18 years (*n* = 84) to evaluate potential heterogeneity in treatment response across skeletal maturity stages. These 1-year intervals (15 –< 16, 16 –< 17, and 17 –< 18 years) were selected to capture the progressive decline in growth potential across late puberty, allowing evaluation of treatment response across distinct stages of skeletal maturity, with the 15 –< 16 group representing the lowest bone age in this range and the 17 –< 18 group representing the highest.

### 2.2. Study Protocol

All patients received once-daily subcutaneous GH at a fixed starting dose of 0.05–0.07 mg/kg/day based on clinical judgment, combined with oral letrozole (Femara^®^, Novartis AG, Stein, Switzerland; 2.5 mg once daily). No systematic dose titration was performed during follow-up. Due to the retrospective study design, quantitative adherence data and longitudinal IGF-1 measurements were not systematically collected, as monitoring focused primarily on growth velocity and safety parameters. Treatment was discontinued upon radiographic confirmation of femoral and tibial physeal closure, growth < 0.7 cm over 3 months, or voluntary withdrawal by guardians or patients. Letrozole was used off-label; written informed consent was obtained from guardians, with assent from patients aged ≥ 12 years, after counseling on potential risks (e.g., estrogen suppression) and monitoring requirements.

Clinical data were retrospectively extracted from medical records, including baseline evaluations and serial follow-up assessments at 3-month intervals.

Anthropometric data included weight (calibrated digital scale; ±0.1 kg) and standing height (Harpenden stadiometer; ±0.1 cm). Body mass index (BMI) was derived as weight (kg)/height (m^2^). Bone age-adjusted standard deviation scores (SDS-BA) for height were calculated using Chinese reference data stratified by BA and sex [17]. MPH was defined as the average of parental heights plus 6.5 cm for males. Annualized height velocity (HV, cm/year) was computed as the difference between final and baseline height divided by the observation period. Pubertal staging followed Tanner criteria [18]. BA was determined from left hand-wrist radiographs using the Greulich-Pyle atlas [19], and PAH was calculated using the Bayley-Pinneau method [20]. Knee epiphyseal status was assessed from anteroposterior and lateral radiographs using the Pyle and Hoerr atlas [21]. Open knee physes were defined as the presence of visible radiolucent physeal plates at the distal femur and/or proximal tibia, indicating incomplete epiphyseal fusion. All bone age radiographs were retrospectively re-read by the same pediatric endocrinologist and the same radiologist, both of whom were blinded to patients’ clinical information, including chronological age, treatment history, and prior bone age reports. Serum IGF-1 levels were quantified using chemiluminescent immunoassay and expressed as age- and sex-adjusted SDS [22].

Baseline assessment covered seven domains: (1) perinatal parameters (birth weight and length); (2) familial growth (parental heights, MPH); (3) anthropometrics (chronological age, height, height SDS-BA, weight, BMI, blood pressure); (4) pubertal development (Tanner stage); (5) skeletal maturation (BA, knee epiphyseal status, PAH); (6) laboratory analyses (metabolic profile, endocrine markers, nutritional indicators, reproductive hormones); and (7) treatment information (GH and letrozole start dates, dosages, and duration).

Longitudinal monitoring consisted of three components: (1) quarterly clinical evaluations, including anthropometrics, pubertal staging, blood pressure, and laboratory tests; (2) biannual radiographic assessments using hand-wrist and knee imaging to track BA progression and physeal closure; and (3) systematic documentation of adverse events, such as acne, radiologically confirmed fractures, and scoliosis (Cobb angle > 10°).

FAH was determined using a multimodal validation protocol. At least 12 months after treatment completion, participants reported their morning height (averaged from three consecutive measurements) via structured telephone interviews, which were cross-verified with the last clinical measurement. FAH was confirmed by either: (1) radiographic evidence of femoral or tibial physeal closure on final knee imaging; or (2) documented HV < 1.0 cm/year over ≥ 12 months of follow-up (self-reported height was accepted for participants unable to attend in-person visits). Cases with a discrepancy > 1.0 cm between self-reported and clinically measured heights were excluded. Ambiguous data were adjudicated by two pediatric endocrinologists, with disagreements resolved by consensus.

### 2.3. Outcomes

Efficacy outcomes included short-term auxological changes (height and annualized HV) and long-term growth outcomes (attained FAH vs. baseline PAH).

Safety outcomes included endocrine and metabolic disorders, musculoskeletal events, and treatment-related adverse events.

### 2.4. Statistical Analysis

Participants were stratified into three BA groups at baseline: 15 ≤ BA < 16, 16 ≤ BA < 17, and 17 ≤ BA < 18. Continuous variables were expressed as mean ± standard deviation (SD) and compared using Student’s *t*-test (for two-group comparisons) or one-way ANOVA (for multi-group comparisons). Categorical variables were expressed as frequency counts (percentages) and evaluated using the Chi-square test or Fisher’s exact test, as appropriate.

Longitudinal changes in height SDS-BA and HV were assessed using linear mixed-effects models with time as a fixed effect and participant-specific random intercepts. Time-by-group interaction terms were introduced to compare the rates of change across BA subgroups; differences in slopes between groups were estimated from these models. Pairwise comparisons of slope differences were performed among the three BA groups. Statistical significance was defined as a two-tailed *p* < 0.05 for all analyses. All statistical procedures were performed using R software (version 4.0.0; R Foundation for Statistical Computing). In total, 95% confidence intervals for mean differences were calculated using the t-distribution; for estimates derived from linear mixed-effects models (e.g., slope differences), CIs were based on the model estimates and their standard errors.

## 3. Results

### 3.1. Study Population

**Figure 1:**
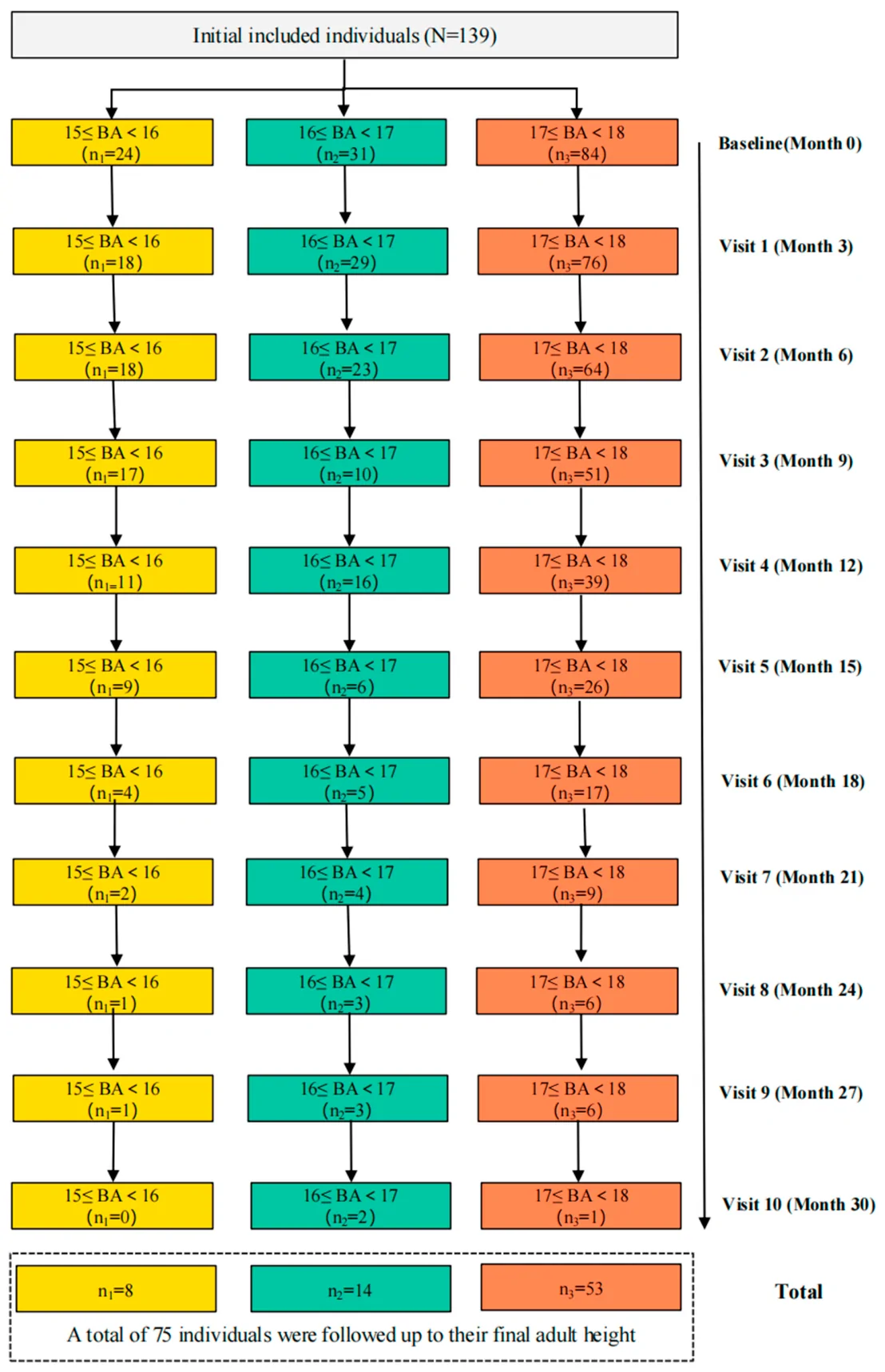
Participant flow diagram illustrating enrollment, stratification, and attrition patterns.

A total of 139 eligible patients were included and stratified by baseline BA into three subgroups: 15 ≤ BA < 16 years (*n* = 24), 16 ≤ BA < 17 years (*n* = 31), and 17 ≤ BA < 18 years (*n* = 84). Follow-up completion rates declined over time: 105 patients (75.5%) completed the 6-month visit, 66 (47.5%) completed the 12-month visit, and 41 (29.5%) completed the 15-month visit, with only three participants remaining at 30 months (Figure 1). Reasons for attrition included voluntary withdrawal (*n* = 94), loss to follow-up (*n* = 16), radiographic confirmation of femoral or tibial physeal closure (*n* = 10), and growth < 0.7 cm over 3 months (*n* = 16). Documented reasons for voluntary withdrawal included financial burden, side-effect concerns, inconvenience of frequent visits, and satisfaction with achieved height. No withdrawals were attributed to severe adverse events. Treatment duration ranged from 3 to 30 months (median 12.0 months; IQR 6.0–15.0).

A total of 75 patients (54.0%) had sufficient follow-up data to determine FAH—defined as having either radiographic evidence of physeal closure or a documented height velocity < 1.0 cm/year over ≥12 months of follow-up, regardless of whether they remained on active treatment throughout the entire observation period. To assess potential selection bias, we compared baseline characteristics between patients who completed follow-up to FAH (*n* = 75) and those who did not (*n* = 64). No significant differences were observed in baseline chronological age (13.81 ± 1.02 vs. 13.69 ± 1.08 years, *p* = 0.27), bone age (16.53 ± 0.72 vs. 16.61 ± 0.81 years, *p* = 0.35), height SDS-BA (−1.40 ± 0.72 vs. −1.39 ± 0.80, *p* = 0.43), or Tanner stage distribution (*p* = 0.57).

### 3.2. Baseline Characteristics

Baseline characteristics are shown in Table 1. The overall mean chronological age (CA) was 13.72 ± 1.01 years, and mean BA was 16.55 ± 0.73 years. The BA/CA ratio was stable across subgroups (1.18–1.22), indicating uniformly advanced skeletal maturation. The mean height SDS-BA was −1.38 ± 0.71, and PAH (164.22 ± 4.37 cm) was consistently lower than MPH (170.25 ± 3.96 cm), with similar height SDS-BA across subgroups. Regarding pubertal status, 30 patients (21.58%) were in Tanner stage III, 74 (53.24%) in stage IV, and 35 (25.18%) in stage V, consistent with mid-to-late puberty. The mean serum IGF-1 level was 400.24 ± 133.96 ng/mL (IGF-1 SDS −0.02 ± 1.40).

### 3.3. Longitudinal Changes in Height SDS-BA and Height Velocity

**Figure 2:**
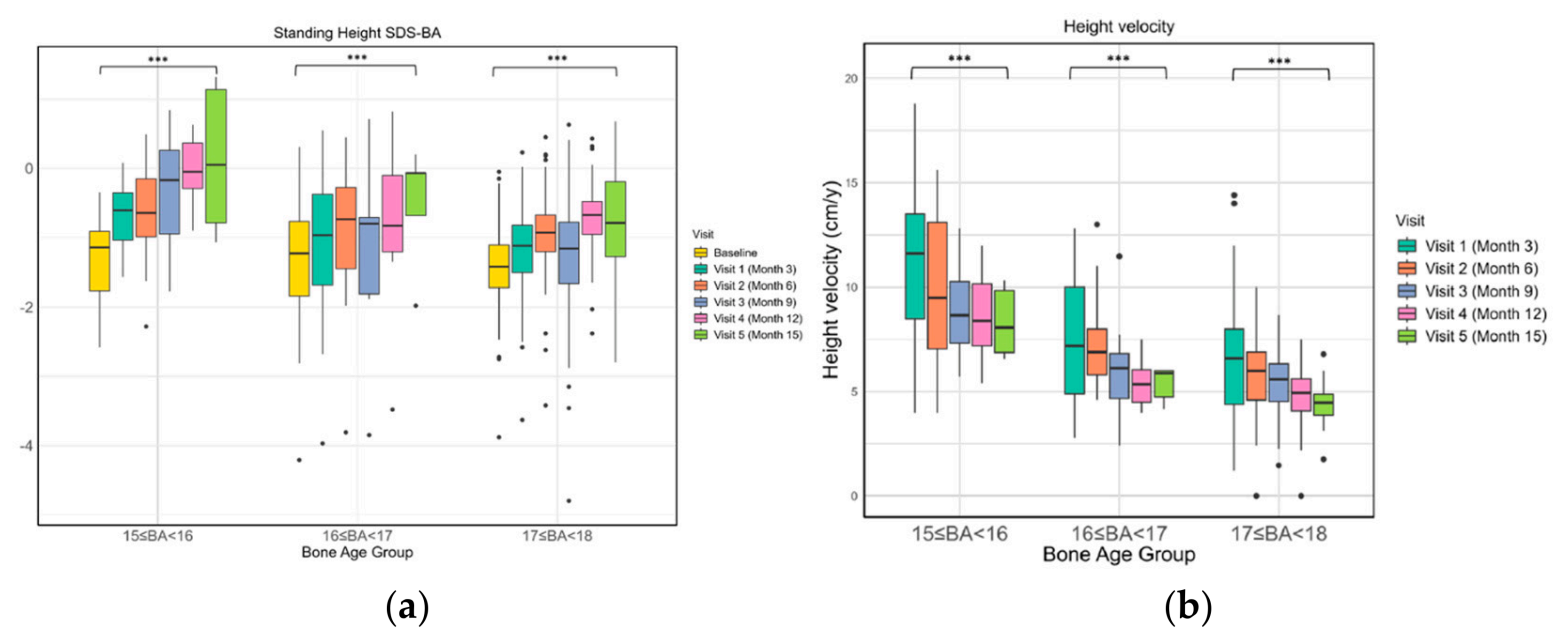
Longitudinal growth response to Letrozole plus GH across bone age subgroups over 15 months. (**a**) Standing height SDS-BA and (**b**) height velocity. Data are shown as boxplots (median, interquartile range, 1.5 × IQR whiskers, outliers as individual points). BA groups: 15 ≤ BA < 16, 16 ≤ BA < 17, 17 ≤ BA < 18. Abbreviations: SDS-BA, bone age-adjusted standard deviation score. *** *p* < 0.001 for time effect within each subgroup.

Figure 2 shows the growth response to letrozole plus GH across BA subgroups over the first 15 months of therapy. Because attrition was substantial beyond month 15, longitudinal analyses were confined to this period. Linear mixed-effects models revealed significant increases in height SDS-BA over time in all three subgroups (15 ≤ BA < 16: F = 64.86, *p* < 0.001; 16 ≤ BA < 17: F = 17.24, *p* < 0.001; 17 ≤ BA < 18: F = 81.48, *p* < 0.001). A time-by-group interaction analysis indicated that the rate of improvement in height SDS-BA was significantly greater in the 15 ≤ BA < 16 group than in the 16 ≤ BA < 17 group (slope difference: 0.133, 95%CI, 0.073–0.194, *p* < 0.001) and the 17 ≤ BA < 18 group (slope difference: 0.142; 95%CI, 0.093–0.191, *p* < 0.001), whereas the two older subgroups did not differ significantly (slope difference: 0.008; 95%CI, −0.040–0.057, *p* = 0.916). Height velocity declined progressively in all three subgroups (15 ≤ BA < 16: F = 13.47, *p* < 0.001; 16 ≤ BA < 17: F = 3.58, *p* = 0.012; 17 ≤ BA < 18: F = 9.97, *p* < 0.001). Pairwise comparisons of the rates of decline showed no significant differences among any of the BA groups (all *p* > 0.05).

### 3.4. Final Adult Height

Among the 75 participants who attained FAH, combined letrozole and GH therapy increased mean FAH by 6.58 ± 3.46 cm relative to baseline PAH (*p* < 0.001). When stratified by BA, height gains showed a significant decreasing trend with advancing BA: 10.04 ± 4.66 cm for BA 15 –< 16 (*n* = 8), 6.30 ± 3.01 cm for BA 16 –< 17 (*n* = 14), and 5.70 ± 2.52 cm for BA 17 –< 18 (*n* = 53) (*p* for trend < 0.001). FAH exceeded MPH only in the BA 15 –< 16 subgroup (175.88 ± 4.12 cm vs. 170.75 ± 1.46 cm, *p* = 0.013), whereas no significant difference was observed in the older subgroups. Height SDS improved from −1.43 ± 0.74 at baseline to −0.31 ± 0.82 at FAH (*p* < 0.001; Table 2).

### 3.5. Safety Outcomes

The incidence of safety endpoints was summarized in Table 3. Endocrine and metabolic disorders included obesity (6.47%), hypertension (2.15%), hyperhomocysteinemia (19.42%), elevated fasting insulin (28.06%), low HDL-C (9.35%), hyperuricemia (35.25%), and subclinical hypothyroidism (2.88%), with no significant differences across BA subgroups (all *p* > 0.05). No diabetes mellitus occurred. Patients with hyperhomocysteinemia received oral folic acid and vitamin B_12_ supplementation, with levels normalizing within 3~6 months in all cases. Musculoskeletal events were rare: one fracture (0.72%) and scoliosis in eight patients (5.76%), predominantly in the 17 ≤ BA < 18 subgroup. Acne was the most common adverse event (46.76%), with a significantly higher incidence in the 17 ≤ BA < 18 group (56.00%) versus the 15 ≤ BA < 16 group (20.83%; *p* = 0.008).

## 4. Discussion

To our knowledge, this study represents the first systematic evaluation of the efficacy and safety of letrozole combined with GH in Chinese males with short stature and advanced bone age (BA ≥ 15 years), based on retrospective data from 139 patients over a decade. It is the largest investigation in this BA range. The results demonstrated that letrozole plus GH significantly improved FAH, with efficacy inversely associated with BA. The 15 ≤ BA < 16 subgroup achieved the greatest height gain (10.04 cm), significantly exceeding gains in the 16 ≤ BA < 17 (6.30 cm) and 17 ≤ BA < 18 subgroups (5.70 cm). In the youngest BA subgroup, FAH not only surpassed baseline PAH but also exceeded MPH, challenging the conventional threshold of BA < 14.5 years for AI plus GH therapy [2], and suggesting that patients with BA ≥ 15 years and open knee physes may retain meaningful growth potential. Although the sample size in this subgroup was small (*n* = 8), the magnitude of height gain was consistent with the longitudinal trajectory observed over the first 15 months of therapy.

Longitudinal analysis over the first 15 months further supported these findings. Although height velocity declined at comparable rates across subgroups, the 15 ≤ BA < 16 group achieved a significantly greater rate of height SDS-BA improvement, reflecting greater residual growth capacity and sustained treatment responsiveness in this age range. The greater height gain observed in this subgroup may be attributable to a longer remaining growth period before epiphyseal closure, rather than to superior treatment responsiveness per se, as height velocity declined at similar rates across all subgroups.

A critical interpretive challenge is the extent to which observed FAH gains reflect treatment effect versus the natural growth trajectory that would have occurred given the use of open knee physes as a selection criterion. The Bayley-Pinneau method, based on wrist BA, may systematically underestimate PAH in patients whose knee epiphyseal maturation lags behind wrist maturation. Consistent with this possibility, the youngest BA subgroup achieved FAH exceeding MPH—a finding that could reflect either a genuine treatment effect, underestimation of baseline growth potential by wrist-based PAH, or both. However, the observation that height gains were largest in the subgroup with the greatest radiographically confirmed growth plate patency, and that the slope of height SDS-BA improvement was significantly greater in this group, lends support to a treatment effect beyond natural progression. Furthermore, the absence of a GH-only or untreated control group precludes determination of the independent contribution of letrozole beyond GH or natural growth, and whether the observed gains primarily reflect selection of patients with greater knee-based residual growth potential rather than a pharmacological effect of aromatase inhibition. Nonetheless, we acknowledge that the absence of a comparator group limits causal attribution, and our findings should be interpreted as hypothesis-generating. The GH dose used in this study (0.05–0.07 mg/kg/day) is higher than standard replacement doses for GH deficiency. However, as all patients received combined therapy, we cannot isolate the independent contribution of GH versus letrozole to the observed growth response—a limitation inherent to the study design.

The overall safety profile was acceptable. No diabetes mellitus or severe endocrine disorders occurred. The most common metabolic abnormalities were hyperuricemia (35.25%), elevated fasting insulin (28.06%), and hyperhomocysteinemia (19.42%), with no significant differences across BA subgroups. Notably, post-treatment homocysteine elevation was observed, a finding not previously reported in this population; the underlying mechanisms remain unclear. The observed elevation may reflect altered one-carbon metabolism, potentially related to estrogen suppression-mediated changes in hepatic enzyme activity, though this remains speculative. Potential effects on folate or vitamin B12 status warrant further study. Elevated insulin levels may reflect physiological insulin resistance in adolescence, compounded by reduced adipose tissue aromatase activity, which promotes insulin resistance [23,24]. Longitudinal controlled studies are needed to determine whether this regimen exacerbates insulin resistance.

Regarding bone health, fracture incidence was low (0.72%), and scoliosis was documented in 5.76% of patients, predominantly in the 17 ≤ BA < 18 subgroup; both findings are consistent with previous reports [11]. However, given that bone health can be influenced by physical activity, external factors, and potential androgen-mediated behavioral changes [25], prospective monitoring of musculoskeletal outcomes remains warranted. Given that estrogen plays a critical role in bone mineral acquisition during puberty, the long-term effects of aromatase inhibition on bone density warrant continued surveillance, particularly in adolescents who may have prolonged treatment duration.

Acne was the most common adverse event (46.76%), with a significantly higher incidence in the 17 ≤ BA < 18 group (56.00%) than in the 15 ≤ BA < 16 group (20.83%; *p* = 0.008). This finding aligns with prior systematic reviews indicating that AI plus GH therapy may induce hyperandrogenic symptoms, likely reflecting higher testosterone levels in older adolescents [26,27].

This study has several limitations. First, as a single-arm retrospective analysis without a placebo control, causal inference is inherently constrained. However, the clinical context—a real-world practice involving an off-label therapy in a population with limited therapeutic options—renders prospective randomization challenging. Our observational findings should therefore be viewed as hypothesis-generating and as providing a foundation for future randomized controlled trials. Furthermore, the use of Bayley-Pinneau-based predicted adult height as a comparator warrants caution, as this method was developed in untreated populations and has not been validated in adolescents receiving aromatase inhibitors, which alter skeletal maturation. Its accuracy in endocrine disorders is also uncertain. Accordingly, the observed PAH-relative gains should be interpreted with these caveats. We also acknowledge that physeal closure is a gradual process, and residual growth potential varies with the degree of physeal patency; our qualitative assessment did not capture this gradation, and future studies using quantitative knee scoring systems may refine residual growth estimation. Additionally, the limited sample size in the FAH analysis (*n* = 75) precluded multivariate regression to assess independent contributions of factors such as treatment duration, age, height z-score, and pubertal stage. Second, the relatively high attrition rate—particularly in the BA 15 –< 16 subgroup, where only 8 of 24 patients were followed to FAH—raises the possibility of selection bias and warrants cautious interpretation of subgroup estimates. Nonetheless, this gain (10.04 cm) was consistent with the significantly greater slope of height SDS-BA improvement observed in the longitudinal analysis. However, given the small sample size in this subgroup, these findings should be interpreted as preliminary and hypothesis-generating, requiring confirmation in larger studies. Furthermore, while the comparison of baseline characteristics between completers and non-completers showed no significant differences, we acknowledge that selection bias due to unmeasured factors cannot be excluded, and therefore attrition-related findings should be interpreted with caution. In addition, long-term skeletal and reproductive safety outcomes were not systematically evaluated in this retrospective study, representing an important limitation that should be addressed in future prospective investigations. Finally, different aromatase inhibitors vary in potency and reversibility [2]; therefore, extrapolation of these findings to other AIs should be undertaken with caution.

## 5. Conclusions

In this retrospective analysis, letrozole plus GH therapy was associated with improved FAH in late-pubertal males with advanced BA and open knee physes. The safety profile appeared acceptable over the follow-up period. However, given the inherent limitations of the study design, these findings require confirmation in prospective randomized trials. In addition, the long-term metabolic and bone safety of this regimen warrants further evaluation.

## Figures and Tables

**Table 1:** Clinical characteristics of study subjects at baseline.

Groups	Total	15 ≤ BA < 16	16 ≤ BA < 17	17 ≤ BA < 18
*n*	139	24	31	84
CA, y	13.72 ± 1.01	12.92 ± 0.93	13.48 ± 0.85	14.04 ± 0.95
BA, y	16.55 ± 0.73	15.12 ± 0.22	16.35 ± 0.23	17.04 ± 0.13
BA/CA	1.21 ± 0.08	1.18 ± 0.09	1.22 ± 0.07	1.22 ± 0.08
Height, cm	163.38 ± 4.47	161.62 ± 4.2	163.58 ± 5.71	163.8 ± 3.94
Height SDS-BA	−1.38 ± 0.71	−1.31 ± 0.61	−1.35 ± 0.92	−1.42 ± 0.65
Weight, kg	58.28 ± 9.69	55.67 ± 10.53	56.14 ± 7.32	59.81 ± 10
BMI, kg/m^2^	21.83 ± 3.54	21.23 ± 3.54	20.96 ± 2.34	22.32 ± 3.85
MPH, cm	170.25 ± 3.96	170.43 ± 3.47	170.9 ± 4.89	169.95 ± 3.71
PAH-BA, cm	164.22 ± 4.37	164.13 ± 4.03	164.41 ± 5.70	164.18 ± 3.94
Tanner stage, *n* (%)				
Stage III	30 (21.58)	12 (50.00)	10 (32.26)	8 (9.52)
Stage IV	74 (53.24)	8 (33.33)	17 (54.84)	49 (58.33)
Stage V	35 (25.18)	4 (16.67)	4 (12.90)	27 (32.14)
IGF-1, ng/mL	400.24 ± 133.96	514.05 ± 153.23	413.38 ± 154.47	362.31 ± 97.24
IGF-1 SDS	−0.02 ± 1.40	1.17 ± 1.60	0.05 ± 1.58	−0.42 ± 1.03

BA, bone age; CA, chronological age; Height SDS-BA, bone age-adjusted standard deviation scores for height; BMI, body mass index; MPH, mid-parental height; IGF-1, insulin-like growth factor-1; PAH-BA, predicted adult height based on bone age. Data are expressed as the mean ± SD or number (%).

**Table 2:** FAH, MPH, and PAH gains across BA subgroups with letrozole plus GH therapy.

Groups	Total	15 ≤ BA < 16	16 ≤ BA < 17	17 ≤ BA < 18
Number	75	8	14	53
PAH, cm	163.94 ± 4.52	164.51 ± 3.50	163.18 ± 6.58	164.06 ± 4.05
MPH, cm	170.50 ± 3.64	170.75 ± 1.46	170.25 ± 5.21	170.53 ± 3.43
FAH, cm	170.81 ± 5.01	175.88 ± 4.12	170.21 ± 6.33	170.20 ± 4.36
Height increment, cm ***, mean ± SD (95%CI)	6.58 ± 3.46 (5.78, 7.38)	10.04 ± 4.66 (6.14, 13.94)	6.30 ± 3.01 (4.56, 8.04)	5.70 ± 2.52 (5.01, 6.39)
Baseline height SDS-BA	−1.43 ± 0.74	−1.25 ± 0.18	−1.54 ± 0.28	−1.43 ± 0.09
FAH SDS	−0.31 ± 0.82	0.52 ± 0.68	−0.41 ± 1.04	−0.41 ± 0.71
*p* (PAH vs. FAH)	<0.001	0.001	0.003	<0.001
*p* (MPH vs. FAH)	0.413	0.013	0.782	0.992
*p* (baseline SDS vs. FAH SDS)	<0.001	<0.001	<0.001	<0.001

BA, bone age; CI, confidence interval; PAH, predicted adult height; MPH, mid-parental height; FAH, final adult height; Height SDS-BA, bone age-adjusted height SDS. *** *p* for trend < 0.001 across BA subgroups.

**Table 3:** Incidence of safety endpoints across BA subgroups.

Safety Endpoints	Total (*n* = 139)	15 ≤ BA < 16 (*n* = 24)	16 ≤ BA < 17 (*n* = 31)	17 ≤ BA < 18 (*n* = 84)	*p*
Endocrine and metabolic disorders					
Obesity, *n*% ^a^	9 (6.47)	3 (12.50)	3 (9.69)	3 (3.57)	0.150 ^#^
Hypertension, *n*% ^b^	3 (2.15)	0	0	3 (3.57)	0.753 ^#^
Hyperhomocysteinemia, *n*% ^c^	27 (19.42)	5 (20.83)	8 (25.81)	14 (16.66)	0.537
Elevated fasting insulin level, *n*% ^d^	39 (28.06)	6 (25.00)	10 (32.26)	23 (27.38)	0.818
Diabetes mellitus, *n*%	0	0	0	0	-
Low HDL-C, *n*% ^e^	13 (9.35)	2 (8.33)	3(9.68)	8(9.52)	1.000 ^#^
Hyperuricemia, *n*% ^f^	49 (35.25)	9 (37.50)	8 (25.81)	32 (38.10)	0.458
Subclinical hypothyroidism, *n*% ^g^	4 (2.88)	1 (4.17)	1 (3.23)	2 (2.38)	0.802 ^#^
Musculoskeletal Events					
Fractures, *n*%	1 (0.72)	0	0	1 (1.19)	1.000 ^#^
Scoliosis, *n*%	8 (5.76)	0	1 (3.23)	7 (8.33)	0.447 ^#^
Other Adverse Events					
Acne, *n*%	65 (46.76)	5 (20.83)	13 (41.94)	47 (56.00) **	0.008

BA, bone age; HDL-C, high-density lipoprotein cholesterol. ^a^ Obesity: defined as body mass index (BMI) ≥ 95th percentile for age and sex per Chinese pediatric growth standards; ^b^ Hypertension: systolic or diastolic blood pressure exceeding the 95th percentile for age, sex, and height, confirmed by ≥3 consecutive measurements; ^c^ Hyperhomocysteinemia: fasting homocysteine ≥ 10 μmol/L; ^d^ Elevated fasting insulin: serum insulin > 25 U/mL after ≥8 h fast; ^e^ Low HDL-C: serum HDL-C < 1.04 mmol/L; ^f^ Hyperuricemia: serum uric acid > 420 μmol/L; ^g^ Subclinical hypothyroidism: thyroid-stimulating hormone > 5 mIU/L. ^#^ Fisher’s exact test; ** 15 ≤ BA < 16 vs. 17 ≤ BA < 18, *p* < 0.01.

## Data Availability

The datasets analyzed for this study are available on request to the primary or corresponding authors.

## Abbreviations

The following abbreviations are used in this manuscript:

AI	Aromatase inhibitor
BA	Bone age
BMI	Body mass index
CA	Chronological age
FAH	Final adult height
GH	Growth hormone
GnRHa	Gonadotropin-releasing hormone analogs
HDL-C	High-density lipoprotein cholesterol
HV	Height velocity
IGF-1	Insulin-like growth factor 1
MPH	Mid-parental height
PAH	Predicted adult height
